# Supplementary material for: A New Method of Preparing Aurone by Marine Actinomycetes and Its Potential Application in Agricultural Fungicides
Source: Molecules. 2022 Dec 20;28(1):17. doi: 10.3390/molecules28010017 (PMC9822012; doi:10.3390/molecules28010017)
Supplement: Supplementary file 1 [file molecules-28-00017-s001.zip › molecules-2022345-supplementary.pdf]

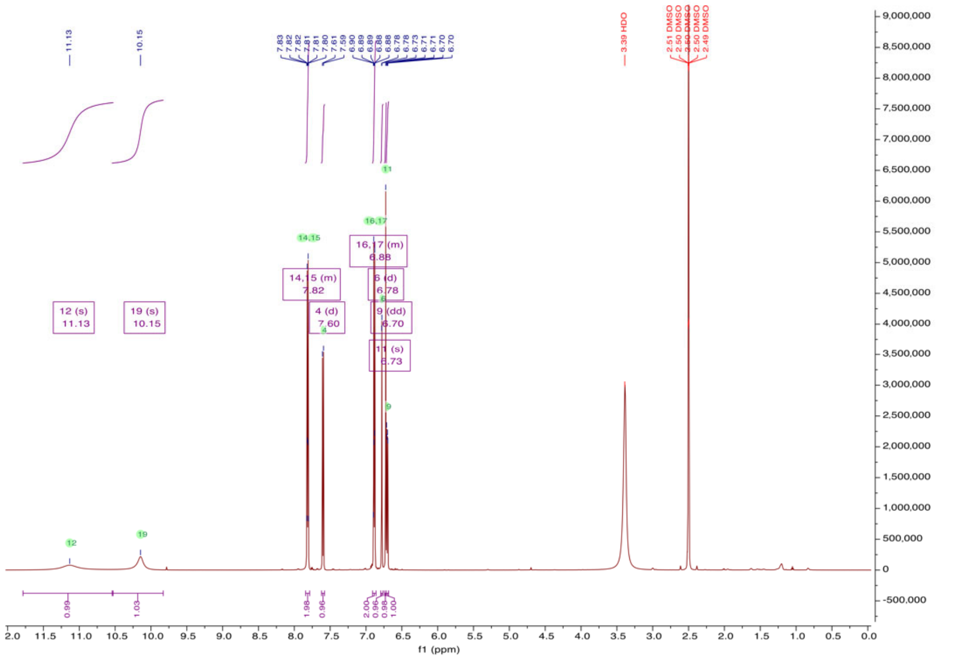

Figure S1. ^{1}H NMR spectrum of the active compound.

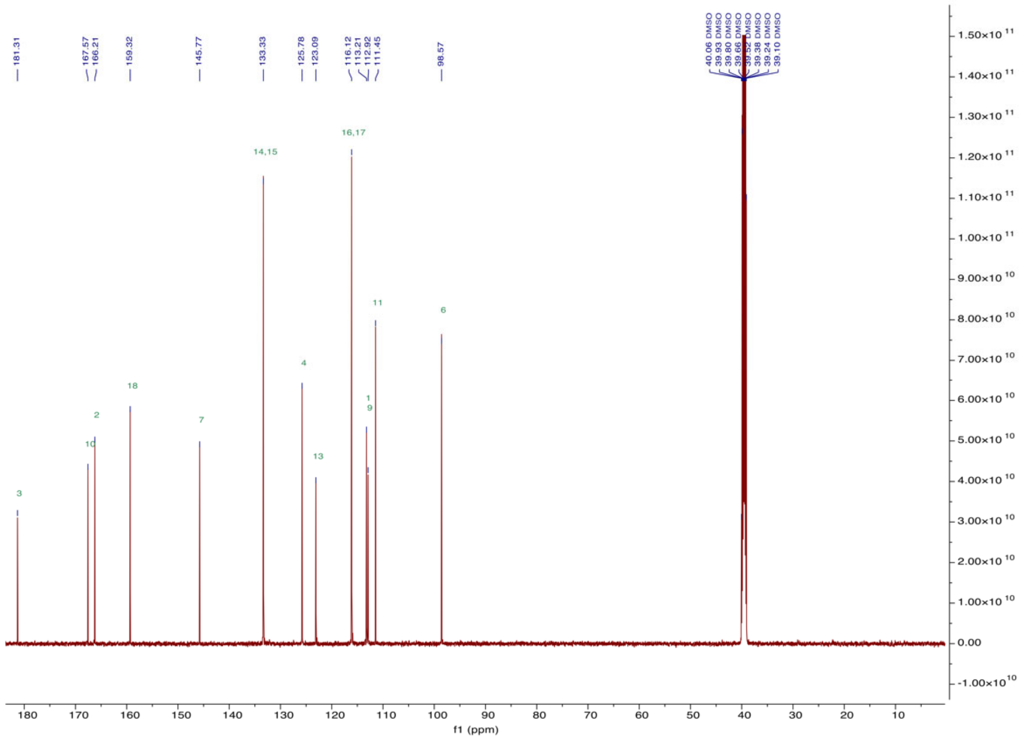

Figure S2. ^{13}C NMR spectrum of the active compound

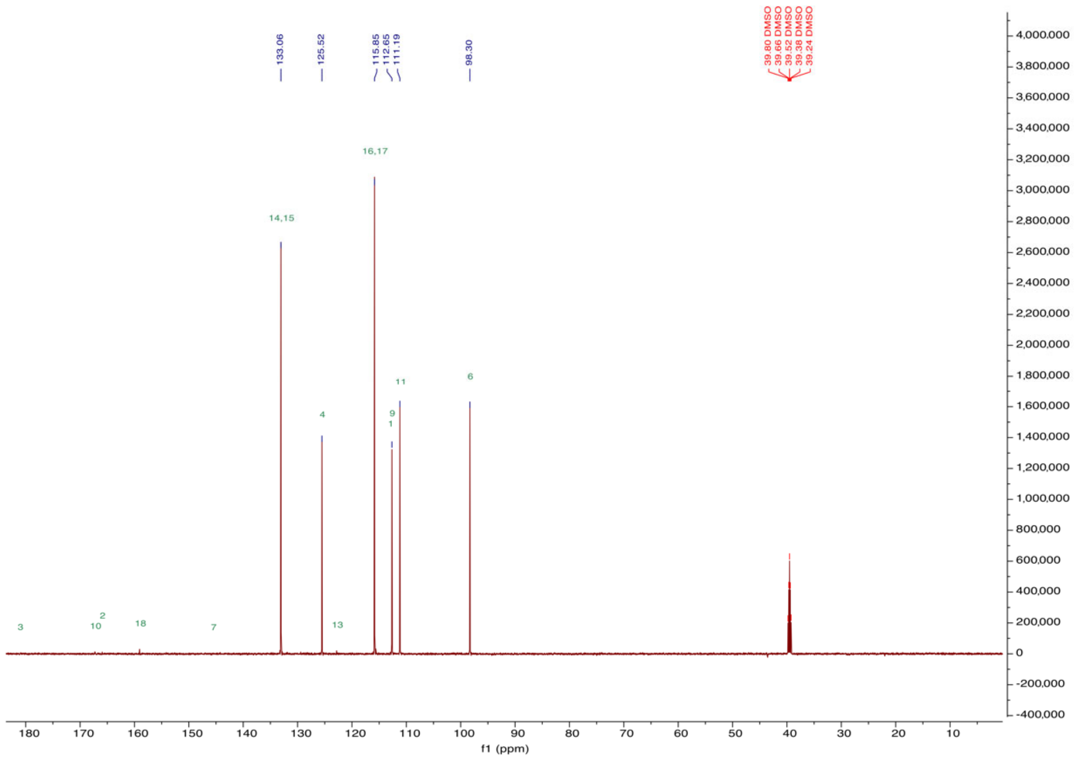

Figure S3. DEPT135 spectrum of the active compound.

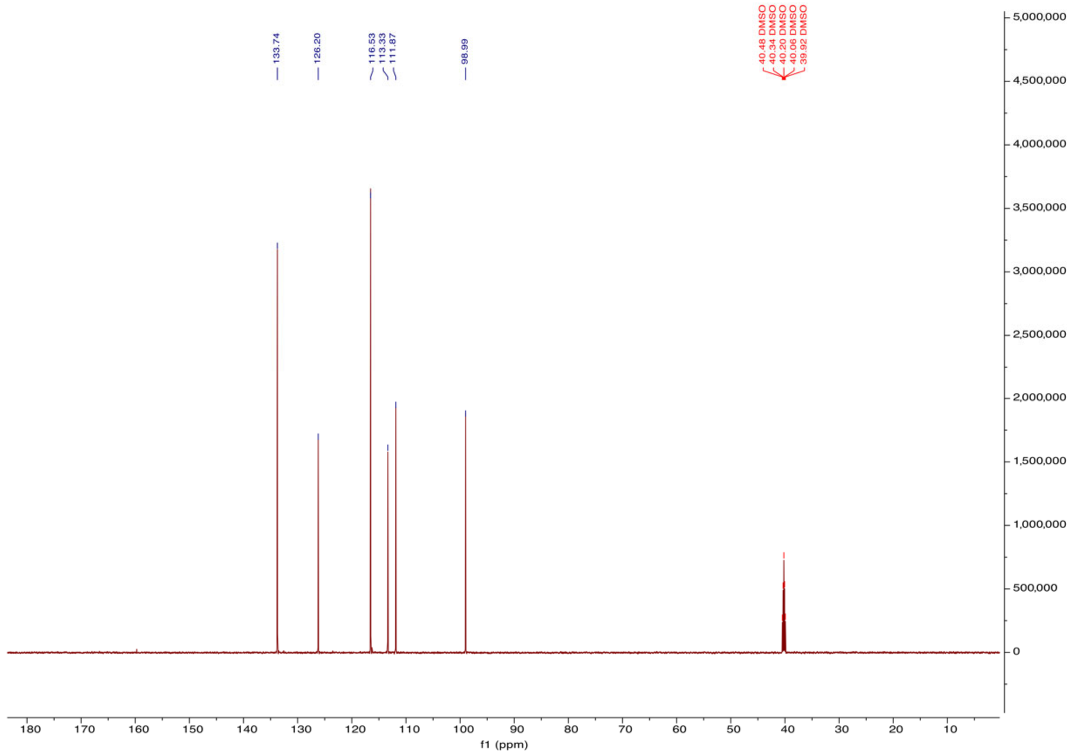

Figure S4. DEPT90 spectrum of the active compound.

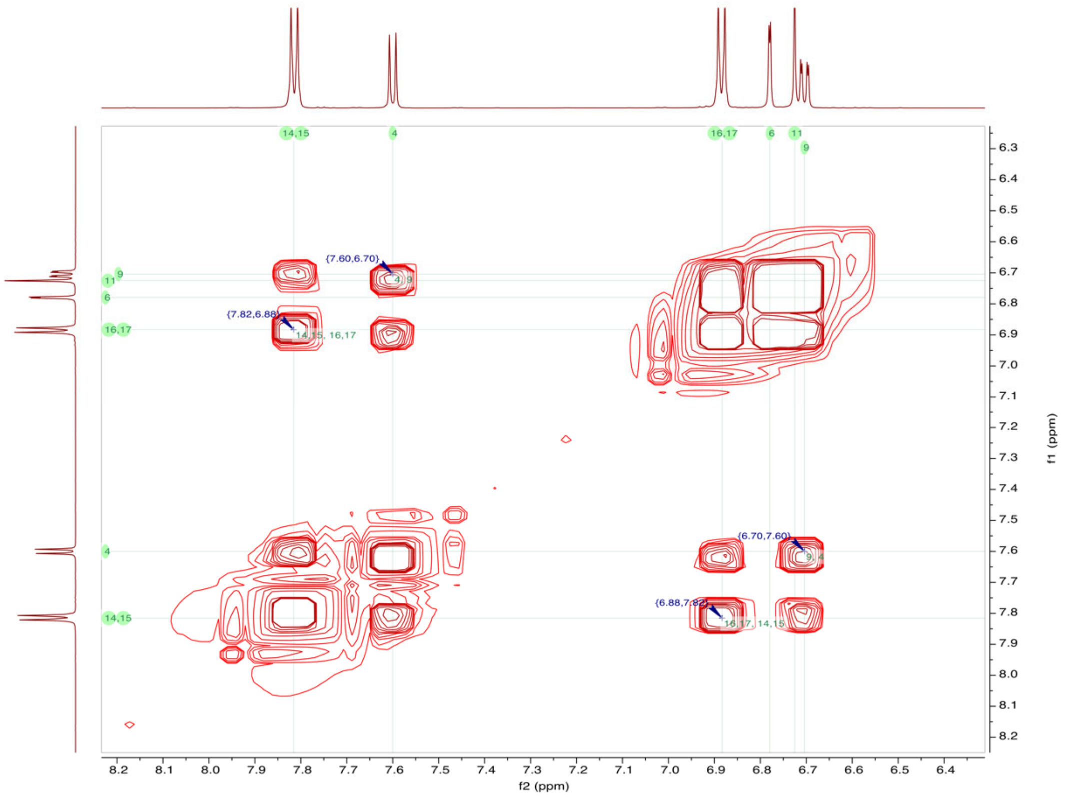

Figure S5. ^{1}H–^{1}H COSY spectrum of the active compound.

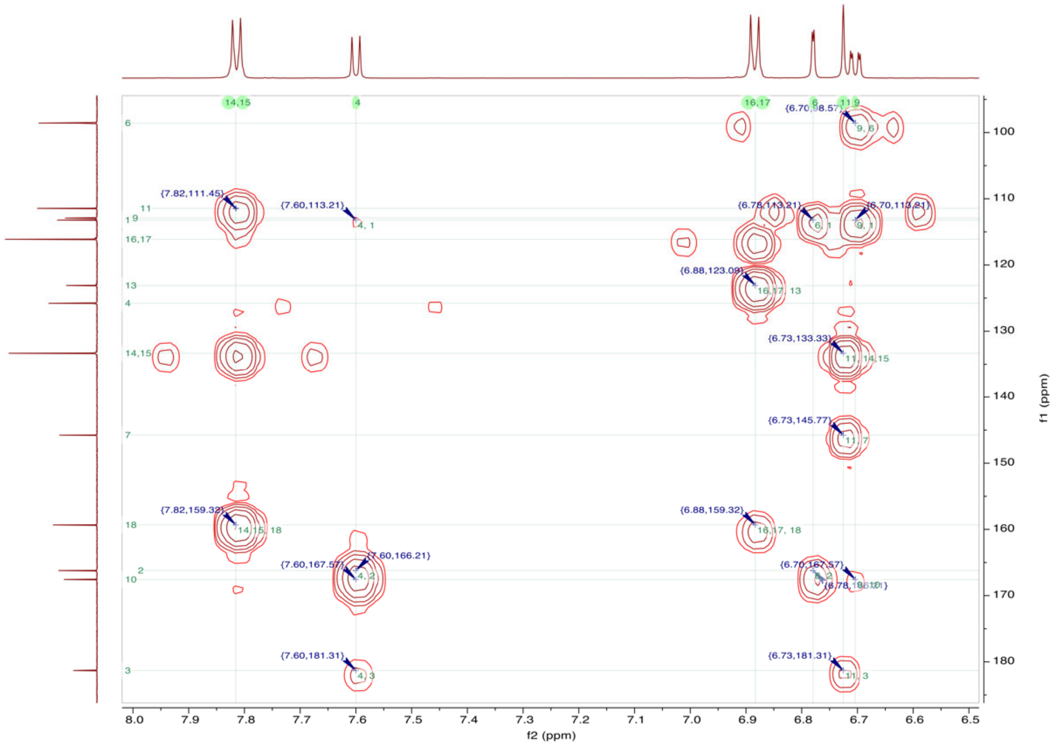

Figure S6. HMBC spectrum of the active compound.
